# Comparative Study of the Structural Features and Electrochemical Properties of Nitrogen-Containing Multi-Walled Carbon Nanotubes after Ion-Beam Irradiation and Hydrochloric Acid Treatment

**DOI:** 10.3390/nano11092163

**Published:** 2021-08-24

**Authors:** Petr M. Korusenko, Sergey N. Nesov, Anna A. Iurchenkova, Ekaterina O. Fedorovskaya, Valery V. Bolotov, Sergey N. Povoroznyuk, Dmitry A. Smirnov, Alexander S. Vinogradov

**Affiliations:** 1 Department of Solid State Electronics, St. Petersburg State University, 7/9 Universitetskaya nab., 199034 Saint Petersburg, Russia; asvinograd@yahoo.de; 2 Department of Physics, Omsk State Technical University, 11 Mira prosp., 644050 Omsk, Russia; nesov@obisp.oscsbras.ru; 3 Laboratory of Physics of Nanomaterials and Heterostructures, Omsk Scientific Center of SB RAS, 15 Karl Marx prosp., 644024 Omsk, Russia; bolotov@obisp.oscsbras.ru (V.V.B.); povorozn@obisp.oscsbras.ru (S.N.P.); 4 Laboratory of Hybrid Materials for Electrochemical Storage Devices, Department of Natural Science, Novosibirsk State University, 2 Pirogova ul., 630090 Novosibirsk, Russia; anna.yurchenkova@yandex.ru; 5 Research Group of Electrochemical Energy Conversion and Storage, Department of Chemistry, School of Chemical Engineering, Aalto University, P.O. Box 16100, FI-00076 Aalto, Finland; fedorovskaya.eo@yandex.ru; 6 Institute of Solid State Physics, Dresden University of Technology, D-01069 Dresden, Germany; wnmw@ya.ru

**Keywords:** nitrogen-containing multi-walled carbon nanotubes, functionalization, oxygen-containing functional groups, pyrrolic and pyridinic nitrogen inclusions, ion beam irradiation, hydrochloric acid treatment, electrochemical behavior, pseudocapacitance, supercapacitors

## Abstract

Using a set of microscopic, spectroscopic, and electrochemical methods, a detailed study of the interrelation between the structural and electrochemical properties of the as-prepared nitrogen-containing multi-walled carbon nanotubes (N-MWCNTs) and their modified derivatives is carried out. It was found that after treatment of nanotubes with hydrochloric acid, their structure is improved by removing amorphous carbon from the outer layers of N-MWCNTs. On the contrary, ion bombardment leads to the formation of vacancy-type structural defects both on the surface and in the bulk of N-MWCNTs. It is shown that the treated nanotubes have an increased specific capacitance (up to 27 F·g^−1^) compared to the as-prepared nanotubes (13 F·g^−1^). This is due to an increase in the redox capacitance. It is associated with the reversible Faraday reactions with the participation of electrochemically active pyridinic and pyrrolic nitrogen inclusions and oxygen-containing functional groups (OCFG). Based on the comparison between cyclic voltammograms of N-MWCNTs treated in HCl and with an ion beam, the peaks on these curves were separated and assigned to specific nitrogen inclusions and OCFGs. It is shown that the rate of redox reactions with the participation of OCFGs is significantly higher than that of reactions with nitrogen inclusions in the pyridinic and pyrrolic forms. Moreover, it was established that treatment of N-MWCNTs in HCl is accompanied by a significant increase in the activity of nitrogen centers, which, in turn, leads to an increase in the rate of redox reactions involving OCFGs. Due to the significant contribution of redox capacitance, the obtained results can be used to develop supercapacitors with increased total specific capacitance.

## 1. Introduction

The development of new materials for electrochemical applications in nanoelectronics and biotechnology is a promising area of modern materials science. One of the coming materials for electrochemical devices such as biological sensors or supercapacitors is multi-walled carbon nanotubes (MWCNTs). This is due to their high strength, structural stability, flexibility, as well as low resistivity. As-prepared nanotubes, when used as a supercapacitor electrode material, usually have a specific capacitance of ~14–30 F·g^−1^ at a potential scan rate of 5 mV·s^−1^, which is accumulated due to the double electric layer (DEL) formation, the so-called electric double layer capacitance (EDLC) [1,2]. In these studies, a 1M aqueous solution of H_2_SO_4_ was used as the electrolyte. Various ways are used to increase the specific capacitance of nanotubes. For example, functionalization of MWCNTs with oxygen-containing functional groups (OCFG) is one of the promising ways to increase the total specific capacitance of a given material to ~240 F·g^−1^ at a potential scan rate of 5 mV·s^−1^ in a 1M aqueous solution of H_2_SO_4_ electrolyte [3]. Moreover, the high characteristics of such nanotubes are associated with energy storage not only by a DEL formed at the electrode-electrolyte interface but also due to redox processes involving functional groups on the surface of MWCNTs. It was shown that several types of the OCFG are mainly involved in energy storage due to redox processes: hydroxyl, carboxyl, and carbonyl groups [4,5,6]. In the literature, reactions with these groups are often given in the following form (Equations (1) and (2)) [7,8]:

(1) $$>C-OH↔>C=O+H^{+}+e^{-}$$

(2) $$—COOH↔-COO^{-}+H^{+}$$

At the same time, it was shown [9] that hydroxyl groups make a significant contribution to improving the capacitive characteristics. Moreover, C—OH groups can also enhance the EDLC due to the improved hydrophilicity of nanotubes [6].

To attach the OCFGs to the surface of nanotubes, various approaches are used based on the creation of defects in their walls by treatment in acids such as H_2_SO_4_, HNO_3_ [10], H_2_SO_4_/HNO_3_ [11], H_2_SO_4_/HCl or oxidizing agents, such as H_2_O_2_ [12], O_3_ [13], and potassium permanganate (KMnO_4_) [14]. As studies [6,15,16] show, when processing nanotubes using chemical methods, various OCFGs are simultaneously attached to the CNT surface in different ratios depending on the oxidizing agent and processing conditions. Concurrently, carrying out selective functionalization with hydroxyl groups is a rather difficult task and requires the use of multistage processes. For example, in the work [17], it was shown that using three-component treatment of MWCNTs with oxidants (H_2_SO_4_, KMnO_4_, H_2_O_2_) together with ultrasonic treatment, it was possible to increase the concentration of –OH groups from 5 to 8 at.%. In another work [18], it was observed that the preparation of carbon nanotubes with hydroxyl groups is possible by primary fluorination followed by the subsequent reaction of these fluorinated carbon nanotubes with diols and glycerol, in the presence of lithium hydroxide. Plasma treatments are more clean, controllable, and reproducible treatment techniques than chemical ones. It was identified in the work [19] that one can influence the concentration of hydroxyl groups attached to the surface of nanotubes by changing the composition of the plasma-forming gas. In this case, the control of the contribution of each type of (DEL and redox) capacitance is possible by choosing the parameters of the MWCNTs functionalization [20].

Another way to increase the total specific capacitance of MWCNTs is to introduce an electrochemically active impurity into the nanotube wall structure, for example, nitrogen atoms, which are capable of reversible redox reactions. According to recent studies [21,22,23], the capacitance of nitrogen-containing MWCNTs (N-MWCNTs) can reach ~20–190 F·g^−1^ at 5 mV·s^−1^ in a 1 M H_2_SO_4_ electrolyte solution. It was established [24] that nitrogen atoms in the walls of these nanotubes can be in pyridinic, pyrrolic, quaternary, and oxidized forms. However, it was shown [25,26,27,28] that only pyridinic and pyrrolic nitrogen can participate in electrochemical reactions (Equations (3)–(5)):
(3)
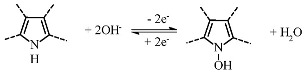

(4)
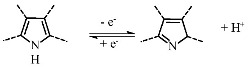

(5)
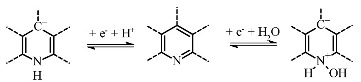



The course and ability of reactions proceeding are strongly dependent on the type of electrolyte used for electrochemical measurements. For example, only pyrrolic nitrogen can participate in redox reactions in an alkaline solution due to the presence of hydrogen bonds, which are easily destroyed (see Equation (3)). This leads to the fact that pyrrolic nitrogen adsorbs the alkaline cation more easily than pyridinic nitrogen, which is inert [26,27]. A different situation is observed in acidic electrolytes. In this environment, both pyrrolic and pyridinic nitrogen provide capacitance through redox reactions involving protons as shown in Equations (4) and (5). Hence, the presence of these types of nitrogen also increases the total capacitance [28,29].

The cyclic voltammetry (CV) method is widely used to analyze the electrochemical behavior of carbon-based materials [30]. In this case, a three-electrode cell consisting of a working electrode, a counter electrode (usually platinum), and a reference electrode [31] is used to carry out a detailed CV analysis of the redox processes, including those involving OCFGs and nitrogen inclusions [32]. In studies [33,34], redox transformations of carbonyl groups and pyridine nitrogen were observed at charge/discharge potentials of 260/100 mV and 825/175 mV, respectively. However, there are few works [22,32,34] in which the potentials of the redox reactions involving OCFGs and nitrogen inclusions are considered simultaneously.

The aim of this work is to study the properties of treated N-MWCNTs to clarify the role of the structural imperfection, the nitrogen inclusions, and hydroxyl groups in the formation of the total specific capacitance of the material. For this, two types of N-MWCNTs samples were prepared: (i) the nanotubes functionalized with hydroxyl groups with a low content of nitrogen inclusions which were prepared in the course of ion-beam irradiation, and (ii) the nanotubes with a high content of electrochemically active nitrogen inclusions and a small amount of OCFGs that were prepared by treatment in HCl. To characterize N-MWCNTs, we used high-resolution transmission electron microscopy (HRTEM), Raman spectroscopy, X-ray photoelectron spectroscopy (XPS), near-edge X-ray absorption fine structure (NEXAFS), the CV method, and electrochemical impedance spectroscopy (EIS).

## 2. Materials and Methods

The synthesis of carbon nanotubes was carried out using the catalytic chemical vapor deposition (CCVD) method in a flow-through gas-phase reactor. Nickel nanopowder obtained by decomposition of nickel oxalate (NiC_2_O_4_) on a quartz substrate was used as a catalyst for the growth of carbon nanotubes. Acetonitrile was used as the precursor for the synthesis of N-MWCNTs. The synthesis temperature and time were 800 °C and one hour, respectively.

Acid treatment of nanotubes was carried out as follows. As-prepared N-MWCNTs (0.25 g) were added to 50 mL of 15% HCl solution in a round bottom flask. The resulting mixture was treated in an ultrasonic water bath for 60 min. The samples were then washed with deionized water and filtered until neutral pH was reached. At the last stage, the nanotubes were dried in an electric tube furnace for 12 h at a temperature of 80 °C.

Ion-beam treatment of nanotubes was conducted using a high-dose ion implanter. N-MWCNTs were irradiated with a continuous beam of argon ions with an average ion energy of 5 keV and a fluence of 1 × 10^16^–5.5 × 10^16^ ion·cm^−2^, followed by exposure of the samples in the implanter chamber in a humid atmosphere overnight.

The structure of nanotubes was characterized by HRTEM using a JEOL JEM 2200FS transmission electron microscope. HRTEM images were measured in bright field mode at an accelerating voltage of 200 kV.

The degree of imperfection of the structure of carbon nanotubes was assessed using Raman spectroscopy. Raman spectra were recorded in quasi-inverse scattering geometry at room temperature with a T64000 spectrometer (HORIBA Jobin Yvon, Longjumeau, France). These spectra were excited by radiation from an Ar^+^ laser with a wavelength of 514.5 nm and a silicon array of photodetectors cooled with liquid nitrogen was used as a detector. The G and D bands were fitted using a Lorentzian function with the application of the XPSPEAK 4.1 software.

Samples for the XPS and NEXAFS measurements were prepared in the following way. A copper tape was glued to the surface of the sample holder, onto which nanotube powder was then poured. For good fixation to the sample holder, the nanotubes were pressed by hand through filter paper. Before measurements, all samples were kept for 12 h in an ultrahigh vacuum and then heated at 90 °C to remove moisture.

The local atomic structure and chemical states of carbon and nitrogen atoms were studied by XPS and NEXAFS methods using monochromatic synchrotron radiation and facilities of the Russian–German beamline at the BESSY II electron storage ring (Helmholtz-Zentrum Berlin, Berlin, Germany) [35]. The XPS spectra of the C 1s and N 1s core levels, as well as survey spectra, were recorded using a Phoibos 150 hemispherical analyzer (Specs GmbH, Berlin, Germany) at an exciting photon energy of 400, 500, and 850 eV with a total energy resolution of 500, 600, and 1300 meV, respectively. The binding energy scale was calibrated against the Au 4f_7/2_ core-level peak and the Fermi level measured from clean gold foil. When measuring the core-level and survey spectra, the analyzer operated in the fixed transmission mode with a pass energy of 20 and 50 eV, respectively. C 1s and N 1s core-level peaks were analyzed by peak fitting applying Gaussian–Lorentzian products at a 70:30 ratio using Casa XPS 2.3.16 software [36]. The Doniach–Sunjic (DS) function was applied for the line shape of the C 1s spectra [37]. The C1s NEXAFS spectra were measured in the total electron yield (TEY) mode by detecting the sample drain current as the energy of the photons incident on the sample changed. The C 1s absorption spectra were normalized to the incident photon flux from a clean gold foil surface mounted on a manipulator holder. When measuring the absorption spectra, the incidence angle of photons was 45°, and the resolution of the monochromator in the vicinity of C 1s absorption edge was ∼70 meV. The photon energy in the range of 70–500 eV was calibrated using Au4f_7/2_ photoelectron (PE) spectra measured with radiation reflected by a diffraction grating in the first and second diffraction orders [38].

To prepare an electrode, a sample weighing 3–4 mg was taken from the analyzed material and transferred to an agate mortar. A few drops of ethanol and 1 μL of a binder (40% aqueous solution of F4D fluoroplastic) were added to the material and mixed in the mortar. It was then transferred to a glass surface and rolled out to form a dense black film. The film was rolled out until it separated from the surface of the glass. The material prepared in this way was a working electrode, and its size was approximately 1 cm^2^. The study of the electrochemical characteristics of the materials was performed by the CV method using the potentiostat/galvanostat P40-X (Electrochemical Instruments, Chernogolovka, Russia). All measurements were accomplished in a three-electrode cell. A 1M aqueous solution of H_2_SO_4_ was used as an electrolyte. A platinum foil was applied as a counter electrode, and Ag|AgCl electrode was used as a reference electrode. Measurements were carried out in the range from 0 to 1000 mV at scan rates of 5, 20, 40, 80, 120 and returning to a rate of 5 mV·s^−1^. The total specific capacitance (*C*) of the electrode was determined by the formula *C* = *A*/(Δ*U*·*V*·*m*), where *A* is the square of the positive curve, Δ*U* is the potential window, *V* is the scan rate, and *m* is the mass of the carbon nanomaterial. Detailed information about electrochemical measurements is given in [39]. The EIS method was performed to study the diffusion processes on the surface of the electrodes before and after long cycling. The measurements were fulfilled at a bias potential equal to zero and over a frequency range from 1 MHz to 0.5 Hz.

## 3. Results and Discussions

**Figure 1:**
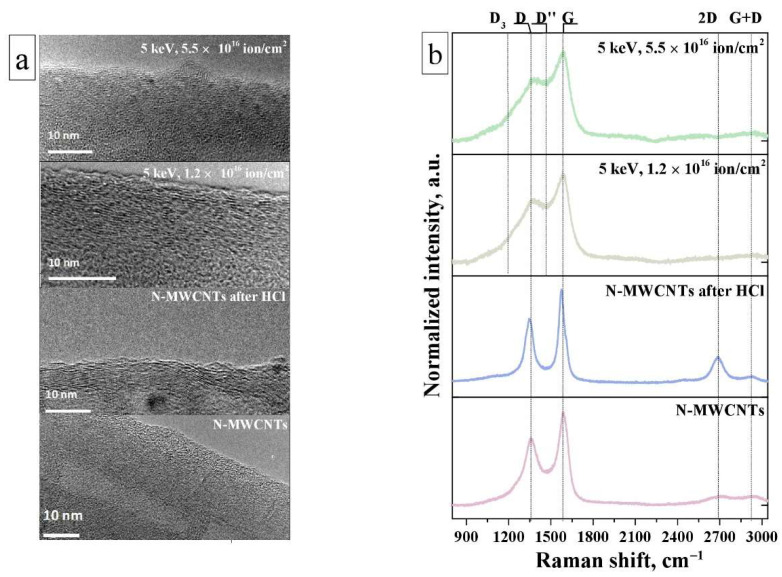
(**a**) HRTEM images and (**b**) Raman spectra of N-MWCNTs before and after treatments.

Analysis of the TEM image of the as-prepared nanotubes shows that they have a bamboo-like structure consist of alternating graphene layers (Figure 1a and Figure S1a). The inner part of the nanotubes has cavities, separated from each other by the inner walls. As can be seen, a layer of amorphous carbon is present on the surface of the as-prepared nanotubes (Figure 1a). In the N-MWCNTs sample after treatment in HCl, no amorphous carbon is observed, which indicates its removal by acid. In addition, the acid treatment also leads to the leaching of the catalyst particles from nanotubes tips (Figure S1b). However, as a result of such treatment, the outermost graphene layers are damaged.

The treatment of nanotubes with an ion beam leads to significant changes in their structure in comparison with the as-prepared N-MWCNTs: there is a significant disordering of graphene layers, their rupture with the formation of graphene fragments, differently oriented relative to the nanotube axis (Figure 1a). This observation means that during irradiation various structural defects of the vacancy type are formed: point and extended defects of more complex shapes, such as (i) vacancy clusters, which are formed by removing several carbon atoms, (ii) combinations of several mono- and divacancies. At the same time, with an increase in the ion beam fluence, more serious damage to the structure of N-MWCNTs is observed (Figure 1a and Figure S1c).

Figure 1b shows the Raman spectra. The spectra of all samples exhibit two intense D and G bands [40]. The G band at ~1590 cm^−1^ is present in all graphite materials and corresponds to the vibrational E_2g_ mode in the graphene planes of the sp^2^-hybridized carbon atoms [40]. The D (~1345 cm^−1^) band is associated with an inactive mode (e.g., A_1g_), which goes into active form a decrease in the symmetry of the carbon lattice due to the presence of structural defects [40,41]. In addition, in the Raman spectra of some samples, there are bands of the second order: a 2D band at 2690 cm^−1^, which is the overtone mode of the D band, and a band at 2930 cm^−1^, which is a combination of D and G bands. As can be seen from Figure 1b and the data in Table 1, the G band shifts towards low wavenumbers (red shift) as a result of treatment in HCl, while for Ar^+^-irradiated samples, this band shifts in the opposite direction relative to its position in the spectrum of as-prepared nanotubes. It was shown in work [42] that the blue shift of the G band depends on the levels of oxidation of the graphene. Thus, we believe that its shift (see Figure S2) is caused by changes in the electronic structure of nanotubes due to the fixation of OCFGs on their surface after ion-beam exposure. At the same time, the red shift of the G band for the nanotubes after HCl (see Figure S2) is probably associated with a decrease in the concentration of nitrogen atoms embedded in nanotubes walls [43] or activation of pyridinic and pyrrolic nitrogen inclusions as a result of their additional protonation. The largest shift (up to 18 cm^−1^) observed for the D band in the treated samples indicates the appearance of new types of disorder in nanotubes as compared to the as-prepared N-MWCNTs (Figure 1, Table 1) [43]. These types of disordering include the formation of vacancies, vacancy clusters, fixation of OCFGs near these defects, etc. [41,42]. The presence of new defect states in nanotubes is most clearly seen from the examination of the spectra of the samples after ion treatment: two new bands appear at about 1500 and 1200 cm^−1^, which are designated as D″ and D_3_. According to [44], these features can be associated with the presence of amorphous carbon fraction and impurities in the nanotubes, respectively.

Based on the analysis of Raman spectra, an assessment of the change in the defectiveness of the structure of N-MWCNTs after chemical treatment and ion-beam exposure was made. For this, the ratio of the integral intensity of the D band to that of the G band (I_D_/I_G_) was used. The chemical treatment of N-MWCNTs leads to a decrease in the I_D_/I_G_ parameter from 1.3 to 1.1 compared with the as-prepared nanotubes (Table 1, Figure S2). It can be explained by the removal of surface amorphous carbon and reduction of defect number on the surface of nanotubes, which correlates with the TEM data (Figure 1a) [11,45]. Removing it from the surface is also confirmed by an increase in the intensity of the second-order peaks compared with that for the as-prepared N-MWCNTs (Figure 1b). The ion-beam treatment has a contrary effect on I_D_/I_G_ ratio and leads to a significant increase in its value from 1.3 to 3.0 relative to that for the as-prepared N-MWCNTs (Table 1, Figure S2). It indicates an increase in the degree of disordering due to radiation-induced formation of structural defects. The maximum changes are observed upon irradiation with a beam fluence of 5.5 × 10^16^ ion·cm^−2^. It is also possible to calculate the average size of graphene domains (L_a_) in nanographite systems [46] (this equation is given in the footnote to Table 1) using the value of the parameter I_D_/I_G_ determined above and laser line wavelength. It can be seen from Table 1 that after the ion-treatment of nanotubes this value decreases to 5.52 nm in comparison with that for the as-prepared N-MWCNTs (12.73 nm). A decrease in the average size of graphene domains confirms our assumption about the formation of structural defects in the walls of nanotubes after this treatment.

**Figure 2:**
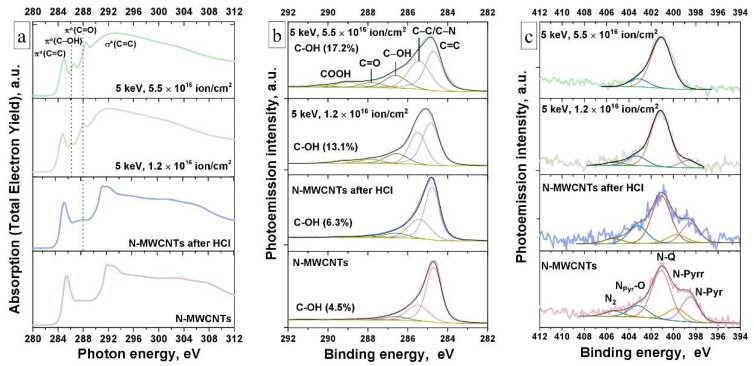
(**a**) C 1s NEXAFS spectra, (**b**) C1s (hν = 400 eV) and (**c**) N1s (hν = 500 eV) PE spectra of N-MWCNTs before and after treatments.

In Figure 2a the C 1s NEXAFS spectra are shown. These spectra are formed by absorption transitions of the C 1s core electrons to the unoccupied states of the π and σ symmetry of the conduction band of MWCNTs as a result of absorption of X-ray quanta of the corresponding energy [41,47]. The presence of intense absorption bands (resonances) π*(C=C) and σ*(C=C) in the C1s NEXAFS spectrum of the as-prepared N-MWCNTs (Figure 2a, curve 1) reflects a high degree of their structural ordering [47]. From Figure 2a (curve 2) it can be seen that treatment of N-MWCNTs in HCl does not lead to changes in the spectrum structure compared to that of the as-prepared nanotubes, except for the appearance of an additional low-energy peak at the photon energy of ~288.5 eV. The appearance of this peak is caused by the C 1s absorption of the OCFGs: carboxyl and carbonyl groups [41]. In the NEXAFS spectra of irradiated N-MWCNTs (Figure 2a, curves 3 and 4), a decrease of π*(C=C) resonance in the intensity and its high-energy shift from ~284.5 to ~284.9 eV are observed. In addition, changes in the shape of the σ*(C=C) resonance and the appearance of new noticeable structures at photon energies of ∼286.4 and ∼288.5 eV are observed. They are caused by transitions of C 1s electrons to unoccupied electronic π*(C—OH) and π*(C=O) states of hydroxyl and carboxyl/carbonyl groups, respectively [41,48]. The noted features of the spectra indicate a significant increase in the defectiveness of the N-MWCNTs structure and the attachment of OCFGs to vacancy clusters formed upon irradiation of nanotubes. In this case, the maximum intensity of the π*(C—OH) absorption band associated with hydroxyl groups on the surface of nanotubes was found for N-MWCNTs irradiated with a beam fluence of 5.5 × 10^16^ ion·cm^−2^.

XPS survey spectra were used to quantify the elemental composition of the surface of all N-MWCNTs (Figure S3a). All spectra exhibit C 1s, N1s, and O1s core-level photoemission (CL PE) lines at binding energies (BE) of ~285, ~401, and ~532 eV, respectively. Using the intensities of these CL PE lines and taking into account their relative sensitivity factors (RSF), the atomic concentrations of all detected elements were calculated, they are summarized in the form of a diagram (Figure S3b). It can be seen in the diagram that, after ion-beam irradiation of N-MWCNTs, the oxygen concentration increases from 1.65 (as-prepared N-MWCNTs) to 16.26 at.% (irradiated with fluence 5.5 × 10^16^ ion·cm^−2^). This is due to the attachment of additional OCFGs to the surface defects of nanotubes during exposure of the latter to humid air in the implanter after irradiation. At the same time, treatment in a HCl does not lead to significant changes in the composition of the sample.

Figure 2b shows the C 1s PE spectra for N-MWCNTs samples before and after treatments. All these spectra are well fitted by five components with maxima at BE of 284.7, 285.4, 286.6, 287.8, and 289 eV, which correspond to C 1s PE lines in C=C (sp^2^), C—C (sp^3^)/C—N, C—OH, C=O and COOH groups, respectively [41,49,50]. In the spectra of the as-prepared and HCl-treated N-MWCNTs, a π-π* shake-up satellite at a BE of ~290 eV is observed, it is usually regarded as a feature of highly ordered graphene-like structures [51]. As can be seen from Figure 2b and the data in Table 2, after the treatment of nanotubes in HCl, a decrease in the intensity of the component responsible for C—C bonds is observed. This finding indicates an increase in the degree of crystallinity of N-MWCNTs due to the removal of amorphous carbon from their surface during HCl treatment. The latter correlates with the above results of a comparative analysis of TEM images, Raman, and NEXAFS spectra (Figure 1 and Figure 2a). In XPS spectra, a slight increase in the C—OH component area to 6.3% for the sample treated with HCl compared with the as-prepared nanotubes (4.5%) may indicate the formation of additional OCFGs attachment sites under the action of acid. Another reason for the increase in the area of this component may be associated with a significant rise in the adsorption activity of neighboring carbon atoms near nitrogen atoms after the removal of amorphous carbon from the N-MWCNTs surface by acid treatment. This assumption is confirmed by a calculation recently performed Dolinskii I.Y. et al., 2018 under the nonorthogonal tight-binding model with the parametrization (NTBM) for the –OH radical adsorbed on the surface of nitrogen-doped graphene in [52]. In the spectra of N-MWCNTs irradiated with an ion beam (Figure 2b), an increase in the relative area of the components corresponding to the C—C, C—OH, C=O, and C—OOH bonds is observed. This observation indicates that ion beam treatment leads to an increase in the degree of imperfection of N-MWCNTs and, as a consequence, to a significant increase in the concentration of OCFGs on their surface. From the data in Table 2, it can be seen that the number of C—OH groups for irradiated N-MWCNTs increases significantly, reaching a maximum value of 17.2% for nanotubes irradiated with a beam fluence of 5.5 × 10^16^ ion·cm^−2^.

The N 1s PE spectra of N-MWCNTs before and after treatments are presented in Figure 2c. The PE spectrum of the as-prepared N-MWCNTs is well fitted by five components with maxima at the BE of 398.4, 399.6, 401.1, 403.3, and 405.4 eV, which correspond to the nitrogen atom in the pyridinic (N-pyr) and pyrrolic (N-pyrr) configurations, as well as in the quaternary (N-Q), pyridinic oxide (N_pyr_—O), and molecular nitrogen (N_2_) forms, respectively [24,41,53,54]. During the N-MWCNTs synthesis, the first four types of nitrogen inclusions are embedded in the walls of nanotubes (Figure S4a), and the last (N_2_) is located between the inner walls and in the cavities of nanotubes [24]. The concentration distribution of nitrogen inclusions (in atomic percent) in N-MWCNTs before and after treatments is shown in Figure S4b.

In the HCl-treated nanotubes, the nitrogen atom is in the same chemical state as in the as-prepared N-MWCNTs, which is clearly seen from the N 1s PE spectra (Figure 2c). At the same time, small changes in the intensity of the pyridinic and pyridinic oxide PE bands are observed in the spectrum of the treated nanotubes. When nanotubes are treated with an ion beam, dramatic changes are observed in the chemical environment of nitrogen atoms (Figure 2c). The relative contributions of all the types of nitrogen inclusions are significantly reduced, except quaternary nitrogen, which is probably more stable under the influence of ion-beam treatment (Figure S4b) [54]. The most changes are observed for a sample irradiated with an ion beam fluence of 5.5 × 10^16^ ion·cm^−2^. The N 1s PE spectrum of this sample shows the presence of nitrogen atoms mainly in the quaternary form (Figure 2c and Figure S4b). The presence of the low-intensity N_pyr_—O component in this spectrum indicates that after ion-beam treatment of nanotubes and their subsequent exposure in a humid environment, partial oxidation of pyridine nitrogen occurs. In addition, a significant decrease in the intensity of the N_2_ component is observed in the N 1s PE spectrum of the N-MWCNTs irradiated with a beam fluence of 1.2 × 10^16^ ion·cm^−2^, as well as its complete disappearance in the spectrum of the nanotubes irradiated with a beam fluence of 5.5 × 10^16^ ion·cm^−2^. This observation means that the ion irradiation of N-MWCNTs leads to the formation of vacancy complexes in their walls, which facilitate the removal of molecular nitrogen (N_2_) from the inter-wall regions and cavities of N-MWCNTs (Figure S4b).

So, it was found above that as-prepared N-MWCNTs contain the pyridinic, pyrrolic, N-Q, and N_pyr_—O nitrogen species and carboxyl, carbonyl, and hydroxyl OCFGs (Figure 2 and Figure S4). For the nitrogen inclusions, it is well known [27,28,29,30] that only pyridinic and pyrrolic nitrogen can participate in electrochemical reactions (see Equations (3)–(5)). In the case of OCFGs, the carboxyl, carbonyl, and hydroxyl groups can be involved in electrochemical redox reactions (see Equations (1) and (2)). In the work [55], it was demonstrated that the oxidation of C—OH with the formation of C=O (Equation (1)) proceeds on the charge curve at the standard potential value of 0.369 V (~0.6 V at pH = 0) vs. Ag|AgCl reference electrode in a 3.5 M aqueous solution of KCl electrolyte. In turn, the reduction process of C=O with the formation of C—OH (Equation (1)) occurs on the discharge curve at the standard potential value of 0.552 V (~0.7 V at pH = 0). The oxidation of C=O, which leads to the formation of –COOH, is observed on the charge curve at a standard potential value of 0.731 V (~0.35 V at pH = 0). The reduction reaction from –COOH to C=O is observed on the discharge curve at 0.306 V (~0.3 V at pH = 0). In paper [56], was shown that additional OCFGs can incorporate in the carbon matrix during electrochemical cycling of the electrode material (Equation (6)):

(6) $$>-C-H\overset{\left[O\right]}{\to }-C-OH\overset{\left[O\right]}{\to }>C=O\overset{\left[O\right]}{\to }-COOH\overset{\left[O\right]}{\to }CO_{2}$$

It should be noted that DEL and redox processes affect the shape of the CV curve. An ideal double-layer capacitor has a rectangular CV shape [7]; a deviation from this shape occurs with the appearance and increase in the number of structural defects (vacancies, holes, edge atoms, and heteroatoms in the structure of material), and/or OCFGs on a material surface. The structural defects directly change the shape of the CV curve, whereas the redox processes resemble peaks on the CV curve.

**Figure 3:**
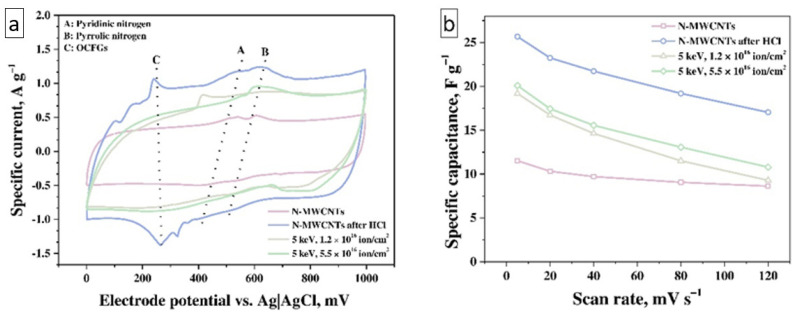
(**a**) The cyclic voltammograms at the scan rate of 40 mV·s^−1^ and (**b**) the specific capacitance in the scan rate range of 5–120 mV·s^−1^ for N-MWCNTs-based electrodes (the 1 M aqueous solution of H_2_SO_4_ electrolyte) before and after treatments.

The CV curves of the as-prepared and treated samples (Figure 3a) show the peaks A, B, and C, which indicate the presence of three types of redox processes on the surface of materials in the 1M aqueous solution of H_2_SO_4_ electrolyte. The smallest contribution from redox processes is observed for as-prepared N-MWCNTs due to a low degree of functionalization: sample contains pyridinic and pyrrolic nitrogen inclusions as well as a small number of carboxyl, carbonyl, and hydroxyl OCFGs (Figure 1, Figure 2, Figures S3 and S4). So, the CV curves of the as-prepared N-MWCNTs sample contain three pairs of redox peaks A_1_–A_2_, B_1_–B_2_, and C_1_–C_2_ on the charge and discharge curves, respectively. To characterize the shape and structure of these CV curves, we will consider the curves obtained at a scan rate of 120 mV·s^−1^, in which the redox peaks are most clearly manifested.

**Figure 4:**
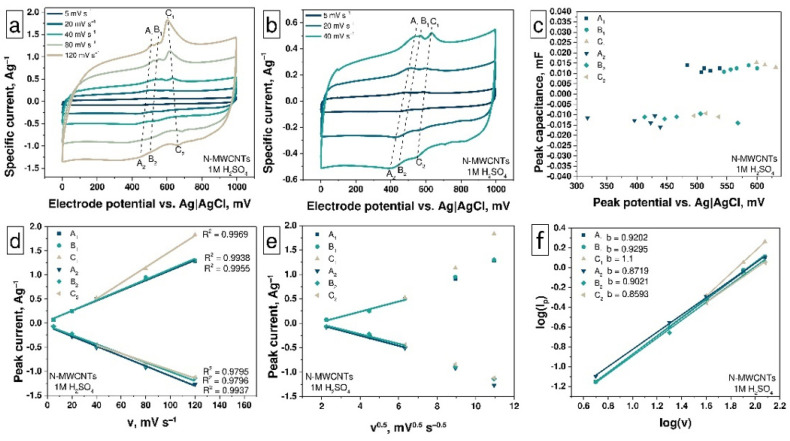
The CV curves at scan rates (v) in the range (**a**) from 5 to 120 mV·s^−1^ and (**b**) from 5 to 40 mV·s^−1^; (**c**) the peak capacitance vs. peak potentials plot; (**d**) scan rate and (**e**) square root of scan rate vs. peak current plots and (**f**) log of scan rate vs. log of peak current plot for as-prepared N-MWCNTs.

The first pair of peaks is represented by the peak A_1_ at 507 mV on the charge curve and the peak A_2_ at 430 mV on the discharge curve (Figure 4a). These peaks correspond to the reversible protonation/deprotonation reaction of pyridinic nitrogen (Equation (5)) [57,58]. The second pair, the peak B_1_ at 545 mV on the charge curve and the peak B_2_ at 506 mV on the discharge curve are attributed to the reversible protonation/deprotonation of pyrrolic nitrogen (see Equation (4)) [59]. Finally, the third pair of peaks, C_1_ and C_2_, which are located at 599 mV and 495 mV on the charge and discharge curves, respectively, are associated with redox reactions involving OCFG. The potential value for the C_1_ peak (0.599 V) is the same as for C–OH (0.6 V) groups oxidation with the formation of C=O groups [55]. It follows from this that the concentration of the C—OH groups should be higher than that of the –COOH groups in this sample. The XPS data from Table 2 confirm that the relative contribution of the C—OH groups (~4.5%) for as-prepared N-MWCNTs is much more than that of the –COOH groups (~1%). The potential value for the C_2_ peak (0.5 V) is in the middle between potentials of C=O reduction reactions with the formation of C–OH (0.7 V) and –COOH (0.3 V). This means that these processes proceed simultaneously and half of the C=O groups are reduced with the formation of C—OH, the other with the formation of –COOH groups. The potentials of all these peaks (A_1_,A_2_; B_1_,B_2_; and C_1_,C_2_) are shifted with a decreasing scan rate (Figure 4a–c): the peaks on the charge curve towards higher potentials and the peaks on discharge curve in the opposite direction in the range of scan rates from 120 to 40 mV·s^−1^. In the range of scan rates from 20 to 5 mV·s^−1^, the nitrogen peaks A_1_, B_1_ (A_2_, B_2_) overlap with peaks C_1_ and C_2_ of OCFGs and are shifted to lower potentials region on the charge curves and to higher-potential regions on the discharge curves (Figure 4b). The overlapping of peaks at scan rates from 20 to 5 mV·s^−1^ and their shifting in the other direction correspond to different kinetics of reactions with nitrogen inclusions and OCFGs. It is also confirmed by the dependencies of the peak current vs. the scan rate (Figure 4d). At high scan rates, the peak current value for OCFGs reactions is much higher than for reactions with nitrogen inclusions. An increase in the current values for A and B peaks and decreasing the current values of the C peaks with the reduction in scan rate value correspond to the fact that the rate of C-reactions higher than that of A- and B-reactions. It may be related to the nature of these processes. The redox reactions involving OCFGs are the surface ones and do not affect the structure of N-MWCNTs, while the reactions involving nitrogen inclusions lead to structural changes in nanotubes. The dependencies of the current value for peaks A–C on the scan rate (Figure 4d) demonstrate a linear behavior in all ranges of scan rates, which is typical for pseudo-capacitive reactions. In turn, the peak current values depend linearly on the square root of the scan rate (Figure 4e) only in the scan rate range from 5 to 40 mV·s^−1^, meaning that the processes are diffusion-controlled in this scan rate range in accordance with the Randles–Sevcik Equation (7) [59,60]:

(7) $$i_{p}=0.4463\cdot v^{1/2}\cdot \frac{z^{3/2}\cdot n^{3/2}\cdot F^{3/2}}{R^{1/2}\cdot T^{1/2}}\cdot c_{0}\cdot D_{av}^{1/2}$$

where *v* is the linear potential scan rate, V·s^−1^; *n* is the number of electrons involved in the electrochemical process; *F* is the Faraday constant, 96.485 C·mol^−1^; *R* is the universal gas constant, 8.314 J·mol^−1^ K^−1^; *T* is the temperature, K; *D_av_* is the averaged chemical diffusion coefficient, cm^−2^·s^−1^; *c*_0_ is the cation initial (maximum) bulk concentration (for the anodic potential scanning) or anion concentration (for the cathodic potential scanning), mol·cm^−3^; *z* is the correction factor.

To confirm the data observed above, the dependencies of the peak current vs. the scan rate on a logarithmic scale were investigated using Equations (8) and (9) [61,62]:

(8) $$i=a\cdot v^{b}$$

(9) log(i)=log(a)+b·log(v)

where the measured current *i* obeys a power-law relationship with the scan rate. Both parameters *a* and *b* are adjustable ones. The b parameter can be calculated by determining the slope of dependence of log(*i*) on log(*v*). So, when parameter *b* is close to 1, the current is predominantly of a capacitive nature. In the case wherein this parameter is equal to 0.5, the current at all potentials changes linearly depending on the square root of the scan rate, which corresponds to an ideal diffusion-controlled process. As shown in Figure 4f, the values of parameter *b* for all of the above reactions are close to 1, indicating their capacitive nature.

The results of measuring the specific capacitance at different scan rates of the electrode potential based on untreated N-MWCNTs (Figure 3b) showed that the specific capacitance of this material decreases from 13 to 10 F·g^−1^ with an increase in the scan rate from 5 to 120 mV·s^−1^.

**Figure 5:**
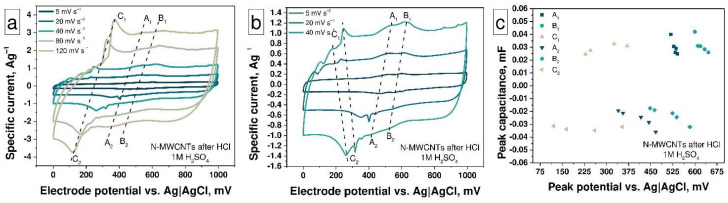
The CV curves at the scan rates in the range (**a**) from 5 to 120 mV·s^−1^ and (**b**) from 5 to 40 mV·s^−1^; (**c**) the peak capacitance vs. peak potentials plot for N-MWCNTs after HCl treatment.

The treatment of N-MWCNTs with HCl (Figure 5a) leads to a change in their functional composition (the amount of OCFGs increases slightly, see Table 2). The interaction between pyridinic or pyrrolic nitrogen inclusions with Cl^-^ anions causes increasing OCFGs activity. The positions of the A_1_, A_2_, and B_1_, B_2_ peaks on the electrode potential scale are the same as for the untreated N-MWCNTs while the position of the peaks C_1_, C_2_ is shifted to the lower-potential region that corresponds to the higher concentration and reaction rate (due to their activation by N atoms in pyrrolic and pyridinic form) of OCFGs in this sample (Figure 4a and Figure 5a). The C (C_1_, C_2_) peaks have the highest specific current in the scan range from 120 to 80 mV·s^−1^ by reason of the fast character of redox processes involving OCFGs and their high concentration (Figure 5a). In this scanning range, the OCFGs peaks located at 400, 350 mV on the charge curve and at 120, 170 mV on the discharge curve, respectively. Decreasing the scan rate results in a splitting of this peak into three peaks, which correspond to the presence of three different OCFGs (Figure 5b). At high scan rates, these peaks overlap, owing to overpotential processes on an electrode surface, while at low scan rates, the overpotential is much lower. Thus, all the peaks are shifted in the same direction at all scan rates (Figure 5c). The behavior of reactions remains the same as in the untreated N-MWCNTs sample, which corresponds to the identical dependencies of the peak current on the scan rate (Figure S5a) and the square root of the scan rate (Figure S5b). The value of the *b* parameter of each reaction is close to 1 and is consistent with the capacitive nature of the reaction (Figure S5c). It should be noted that the *b* parameters for C reactions are the same for as-prepared nanotubes and treated by HCl, while for A and B reactions, they become smaller. This fact manifests itself in the effect of HCl on the nitrogen inclusions and in the activation and boosting of the EDLC. The combination of redox reactions involving OCFG and nitrogen inclusions as well as DEL formation leads to an increase of specific capacitance from 13 F g^−1^ (for as-prepared N-MWCNTs) up to 27 F·g^−1^ at 5 mV·s^−1^ for the nanotubes treated with HCl. At the same time, it was seen above (Figure 3b) that the specific capacitance of this material decreases from 27 to 17 F·g^−1^ with an increase in the scan rate from 5 to 120 mV·s^−1^.

**Figure 6:**
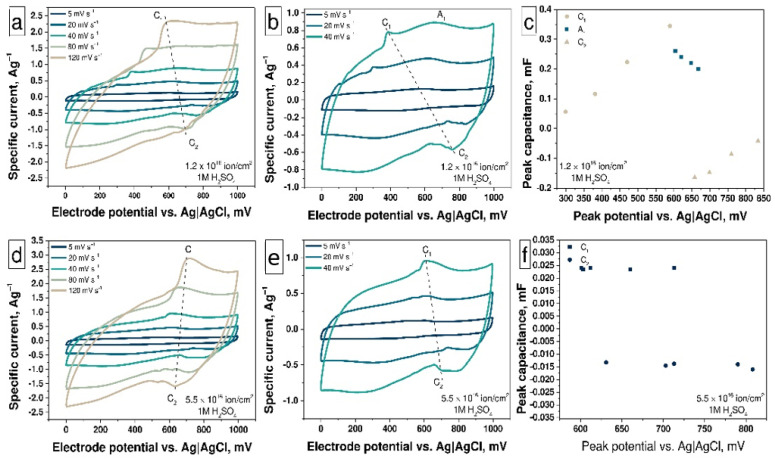
The CV curves at the scan rates in the range from 5 to 120 mV·s^−1^ (**a**,**d**) and from 5 to 40 mV·s^−1^ (**b**,**e**); the peak capacitance vs. peak potentials plot (**c**,**f**) for N-MWCNTs irradiated by ion beam with different fluence.

The ion-beam treatment of N-MWCNTs results in an increase in the concentration of structural defects and OCFGs as well as a decrease in the concentration of pyridinic and pyrrolic nitrogen inclusions (Table 2, Figure 2c and Figure S4). The ion beam treatment with a fluence of 1.2 × 10^16^ ion·cm^−2^ leads to a change in the shape of the CV curve compared with that of as-prepared N-MWCNTs and broadening of the peaks as a result of an increase in the concentration of OCFGs on the N-MWCNTs surface (Figure 3a). Changes in the shape of CV curves correspond to the higher defectiveness of irradiated samples, which was previously confirmed by Raman spectroscopy studies (see Figure 1b). Only C peaks are detected at a scan rate of 120 mV·s^−1^ (see Figure 6a) due to the high concentration of OCFGs and low concentration of pyridinic nitrogen inclusions in this sample (see Figure S4b). At a scan rate of 120 mV·s^−1^, the C_1_ and C_2_ peaks are located at 600 mV on the charge curve and at 700 mV on the discharge curve, respectively. These peak positions coincide with the positions of the corresponding peaks for the redox processes of C—OH groups at pH = 0 in [55]. This is also evidenced by a high concentration of C—OH groups in irradiated samples. The peak A1 appears at scan rates in the range from 80 to 5 mV·s^−1^, which is associated with the low concentration of pyridinic nitrogen inclusions and the splitting of OCFGs peaks (see Figure 6b,c). All these processes demonstrate behavior that is similar to their behavior for the relevant processes in the case of the previous samples (see Figure S6a–c). The capacitance of this sample reaches the value ~19 F·g^−1^.

Furthermore, the ion-beam treatment of N-MWCNTs with the fluence of 5.5 × 10^16^ ion·cm^−2^ leads to the complete disappearance of pyridinic and pyrrolic nitrogen inclusions. Indeed, there are no peaks associated with these types of nitrogen inclusions on CV curves (Figure 6d–f) and only the C peaks are observed at 600 mV on the charge and at 650 mV on the discharge curves (Figure 6f). The behavior of these reactions remains the same (Figure S7a–c). The specific capacitance reaches the value 20 F·g^−1^ for this sample. For both irradiated samples, it can be seen (Figure 3b) that the specific capacitance of this material decreases from ~20 to ~11 F·g^−1^ with an increase in the scan rate from 5 to 120 mV·s^−1^.

To visualize the relationship between the specific capacitance and the degree of defectiveness of nanotubes, depending on the treatment method, the data of electrochemical measurements and Raman studies in the form of a diagram were summarized (see Figure S8).

Investigation of EDLC and Faraday reactions contributions in the total capacitance was carried out using kinetic dependencies of the capacitance on the square root of the scan rate (Equation (10)):

(10) $$C=C_{v=\infty }+\frac{\alpha }{m\cdot \Delta U}\cdot v^{-1/2}\;\mathrm{and}\;C^{-1}=C_{v=0}^{-1}+m\cdot \Delta U\cdot \alpha \cdot v^{1/2}$$

where *C* is the total capacitance, $C_{v=\infty }$ and $C_{v=0}$ are the EDLC and the maximal capacitance, α is a coefficient of electron transport (α can be easily calculated as the square root of multiplication c*d, where *c* = $\frac{\alpha }{m\cdot \Delta U}$ and *d* = m·ΔU·α).

**Figure 7:**
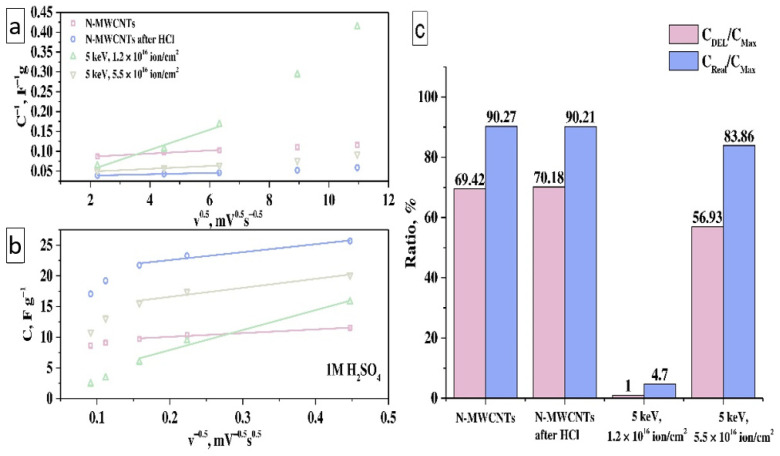
The dependence of the reciprocal of the total capacitance (C^−1^, F·g^−1^) on the square root of the scan rate (v^0.5^, mV^0.5^·s^−0.5^) (**a**) and the total capacitance (C, F·g^−1^) on the reciprocal of the square root of the scan rate (v^−0.5^, mV^−0.5^·s^0.5^) (**b**), the ratios of the double electric layer capacitance (C_DEL_) and the experimental capacitance (C_Real_) to the total theoretical capacitance (C_Max_) (**c**) for electrodes based on N-MWCNTs before and after treatments.

Based on Equation (10), the dependences of C vs. v^0.5^ and C^−1^ vs. v^−0.5^ were obtained, ratios of DEL capacitance to maximal theoretical capacitance and real capacitance to theoretical one were calculated (Figure 7a,b). It was found that as-prepared N-MWCNTs and N-MWCNTs after HCl treatment have the highest EDLC contribution in the total theoretical capacitance C_Max_ (Figure 7c) ~70%. In turn, the experimental value of the specific capacitance C_Real_ for these samples is close to the maximum theoretical value at a scan rate of ~5 mV·s^−1^ and is about ~90%. The contribution of EDLC to the total capacitance decreases from ~70% (for as-prepared N-MWCNTs) to ~1% and ~57% for irradiated N-MWCNTs with a beam fluence of 1.2 × 10^16^ ion·cm^−2^ and 5.5 × 10^16^ ion·cm^−2^, respectively. Similar dependencies of the ratio of the experimental capacitance to the theoretical one (C_Real_/C_Max_) for these samples are ~5% and ~84% for samples N-MWCNTs with a beam fluence of 1.2 × 10^16^ ion·cm^−2^ and 5.5 × 10^16^ ion·cm^−2^, respectively. The decrease in EDLC value after treatment by ion beam occurs due to a reduction in the concentration of pyridinic and pyrrolic nitrogen [25,28,29].

**Figure 8:**
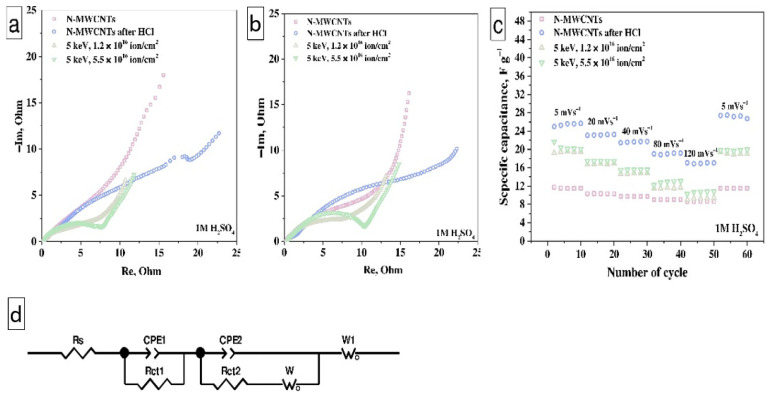
Nyquist plot before (**a**) and after cycling (60 cycles) (**b**), the cycling at different scan rates (**c**), the equivalent circuit (**d**) for electrodes based on N-MWCNTs before and after treatments.

Figure 8 shows the EIS data and an equivalent circuit for the electrodes before and after cycling. The EIS curves of all samples consist of two semicircles; the first one is typical for DEL formation, the second one for redox processes proceeding (Figure 8a). These semicircles overlap with the formation of one broad depressed semicircle in the EIS spectra of samples. The semicircle deformation indicates the contribution of charge transfer resistance, DEL capacitance, and redox processes. The straight line in the low-frequencies region is typical for Warburg impedance, which corresponds to the diffusion of electrolyte ions from materials surface to electrolyte. An equivalent circuit was proposed to describe this system (Figure 8d) [63]. In this circuit, the *Rs* element includes equivalent resistance (a combination of electrolyte, material, and contact resistance with a platinum current collector). This resistance is in the range from 0.23 to 0.35 Ohms and indicates a low voltage drop across all working electrodes before and after cycling. The combination of *CPE*_1_ and *R_ct_*_1_ elements describe the first semicircle. The *CPE*_1_ and *R_ct_*_1_ elements are DEL capacitance and resistance, respectively [64]. The parallel combination of *CPE*_2_ with *R_ct_*_2_ and *W* (Nernst diffusion) describe the second semicircle and is typical of the Faradaic impedance [59,65]. It should be noted that for the electrodes based on N-MWCNT treated with hydrochloric acid and irradiated with an ion beam, an increase in the second circle in comparison with the electrode based on the as-prepared N-MWCNTs is observed. It means that the contribution of Faradaic impedance in the EIS depends on the concentration of the electrochemically active nitrogen inclusions and the OCFG in the samples. In this case, the increase in the radius of the semicircle after cycling occurs due to the deeper penetration of electrolyte ions into the porous structure of the electrodes (Figure 8b). The cycling of samples at different scan rates is represented in Figure 8c. The linear dependencies of capacitance on the number of cycles correspond to the reversible nature of redox reactions. Thus, this result indicates that the treated N-MWCNTs have stable characteristics after cycling and can be used as a material for supercapacitor electrodes.

## 4. Conclusions

Using a set of microscopic, spectroscopic, and electrochemical methods, a detailed study of the interrelation between the structural and electrochemical properties of the as-prepared nitrogen-containing multi-walled carbon nanotubes (N-MWCNTs) and their modified derivatives is carried out.

It was found that after treatment of nanotubes in HCl, their structure is improved due to the removal of amorphous carbon from the surface of N-MWCNTs. However, ion bombardment, on the contrary, leads to deterioration of the structure of nanotubes due to the formation of vacancy-type structural defects both on the surface and in the bulk of the N-MWCNTs. It was established that as a result of N-MWCNT irradiation, the average size of graphene sp^2^ domains decreases from 12.73 to 5.52 nm, which is explained by the rupture of graphene layers (walls) of nanotubes with the formation of graphene fragments oriented differently relative to the nanotube axis. It is shown that the selective attachment of hydroxyl groups to defects on the surface of nanotubes is observed when nanotubes are treated with an ion beam, and their concentration increases with increasing beam fluence.

It was demonstrated that the treated nanotubes have an increased specific capacitance (up to 27 F·g^−1^) relative to the as-prepared nanotubes (13 F·g^−1^). This is caused by the significant contribution of the redox capacitance to the total one due to the occurrence of reversible reactions involving the electrochemically active pyridinic and pyrrolic nitrogen inclusions and the OCFGs. A comparative analysis of the cyclic voltammograms for N-MWCNT functionalized mainly with hydroxyl groups and N-MWCNT after treatment in HCl made it possible to separate the peaks onto the CV curves and associate them with the specific nitrogen inclusions and the OCFGs. Based on the analysis of the kinetic dependences of the peak current on the potential scan rate, it was found that the rates of these reactions are different. Thus, the redox reactions involving the nitrogen inclusions are slow and their rates increase with decreasing the scan rate, whereas the redox reactions involving the OCFGs are fast and show the greatest contribution at high scan rates. We believe that this is due to the fact that the redox reactions involving the OCFGs are the surface reactions and do not affect the structure of N-MWCNTs, while those involving nitrogen inclusions lead to structural changes in nanotubes. Stability tests of the samples under study showed that after cycling, the characteristics of the treated nanotubes remain at the same level, which indicates the reversible nature of reactions involving the nitrogen inclusions and the OCFGs. Obviously, the new results obtained can be used to develop supercapacitors with an increased total specific capacitance due to the significant contribution of redox capacitance. In addition, we believe that the presence of a high concentration of C—OH groups on the surface of nanotubes irradiated with an ion beam may be used for the formation of Me_x_O_y_@N-MWCNTs (Me = metal) composites with high interfacial adhesion.

## Figures and Tables

**Table 1:** D and G bands position and average crystallite size of the graphene sp^2^-carbon domains from the Raman spectra for the N-MWCNTs before and after treatments.

Sample	G, cm^−1^	D, cm^−1^	Integral Intensities Ratio I_D_/I_G_	L_a_^1^, nm
N-MWCNTs	1587	1359	1.3	12.73
N-MWCNTs after HCl	1577	1351	1.1	15.05
N-MWCNTs after 1.2 × 10^16^ ion·cm^−2^	1591	1377	2.8	5.90
N-MWCNTs after 5.5 × 10^16^ ion·cm^−2^	1591	1377	3.0	5.52

^1^ L_a_ = [(2.4 × 10^−10^) (λ)^4^]/[I_D_/I_G_], λ is laser line wavelength (514 нм) [46].

**Table 2:** Five-component approximation for C 1s PE spectra.

Sample	[C] Total, at.%	Relative Areas of Components, %				
		[C=C]	[C—C/C—N]	[C—OH]	[C=O]	[COOH]
N-MWCNTs	94.35	69.5	23.1	4.5	1.9	1.0
N-MWCNTs after HCl	95.58	71.4	19.0	6.3	2.3	1.0
N-MWCNTs after 1.2 × 10^16^ ion·cm^−2^	85.37	47.4	31.2	13.1	5.2	3.1
N-MWCNTs after 5.5 × 10^16^ ion·cm^−2^	82.07	40.1	32.0	17.2	5.5	5.2

## Data Availability

The data presented in this study are available on request from the corresponding author.

## Supplementary Materials

The following are available online at https://www.mdpi.com/article/10.3390/nano11092163/s1, Figure S1: TEM images of as-prepared N-MWCNTs (a) and after treatment in HCl (b) as well as after irradiation by ion beam with φ_2_ = 5.5 × 10^16^ ion·cm^−2^ (c), Figure S2: The G band position vs. I_D_/I_G_ ratio for N-MWCNTs before and after treatments: 1—initial; 2—after HCl; 3—after irradiation by ion beam with φ_1_ = 1.2 × 10^16^ ion·cm^−2^; 4—after irradiation by ion beam with φ_2_ = 5.5 × 10^16^ ion·cm^−2^, Figure S3: (a) Survey PE spectra of N-MWCNTs before and after treatments (hν = 850 eV); (b) diagram of atomic concentration from XPS survey spectra for samples, Figure S4: (a) Types of nitrogen inclusions in the hexagonal lattice of graphene; (b) the distribution of the concentrations of various nitrogen inclusions in N-MWCNTs before and after various treatments, Figure S5: (a) Scan rate and (b) square root of scan rate vs. peak current and (c) log of scan rate vs. log of peak current plots for N-MWCNTs after HCl, Figure S6: (a) Scan rate and (b) square root of scan rate vs. peak current and (c) log of scan rate vs. log of peak current plots for N-MWCNTs irradiated by ion beam with φ_1_ = 1.2 × 10^16^ ion·cm^−2^, Figure S7: (a) Scan rate and (b) square root of scan rate vs. peak current and (c) log of scan rate vs. log of peak current plots for N-MWCNTs irradiated by ion beam with φ_2_ = 5.5 × 10^16^ ion·cm^−2^, Figure S8: The diagram of the specific capacitance of nanotubes vs. their degree of defectiveness from the data of electrochemical measurements and Raman studies.
