# Supplementary material for: Comparative Study of the Structural Features and Electrochemical Properties of Nitrogen-Containing Multi-Walled Carbon Nanotubes after Ion-Beam Irradiation and Hydrochloric Acid Treatment
Source: Nanomaterials (Basel). 2021 Aug 24;11(9):2163. doi: 10.3390/nano11092163 (PMC8471838; doi:10.3390/nano11092163)
Supplement: Supplementary file 1 [file nanomaterials-11-02163-s001.zip › nanomaterials-1346626-supplementary.pdf]

Supplementary information

Article

Comparative Study of the Structural Features and Electrochemical Properties of Nitrogen-Containing Multi-Walled Carbon Nanotubes after Ion-Beam Irradiation and Hydrochloric Acid Treatment

Petr M. Korusenko [1,2,*], Sergey N. Nesov [2,3], Anna A. Iurchenkova [4,5], Ekaterina O. Fedorovskaya [5], Valery V. Bolotov [3], Sergey N. Povoroznyuk [3], Dmitry A. Smirnov [6] and Alexander S. Vinogradov [1]

[1] Department of Solid State Electronics, St. Petersburg State University, 7/9 Universitetskaya nab., 199034 Saint Petersburg, Russia; asvinograd@yahoo.de
[2] Department of Physics, Omsk State Technical University, 11 Mira prosp., 644050 Omsk, Russia; nesov@obisp.oscsbras.ru
[3] Laboratory of Physics of Nanomaterials and Heterostructures, Omsk Scientific Center of SB RAS, 15 Karl Marx prosp., 644024 Omsk, Russia; bolotov@obisp.oscsbras.ru (V.V.B.); povorozn@obisp.oscsbras.ru (S.N.P.)
[4] Laboratory of Hybrid Materials for Electrochemical Storage Devices, Department of Natural Science, Novosibirsk State University, 2 Pirogova ul., 630090 Novosibirsk, Russia; anna.yurchenkova@yandex.ru
[5] Research Group of Electrochemical Energy Conversion and Storage, Department of Chemistry, School of Chemical Engineering, Aalto University, P.O. Box 16100, FI-00076 Aalto, Finland; fedorovskaya.eo@yandex.ru
[6] Institute of Solid State Physics, Dresden University of Technology, D-01069 Dresden, Germany; wnmw@ya.ru
* Correspondence: korusenko_petr@mail.ru

Abbreviations and parameters

N-MWCNTs – nitrogen-containing multi-walled carbon nanotubes
PE – photoemission
φ – ion beam fluence value
hν – exciting photon energy

TEM data

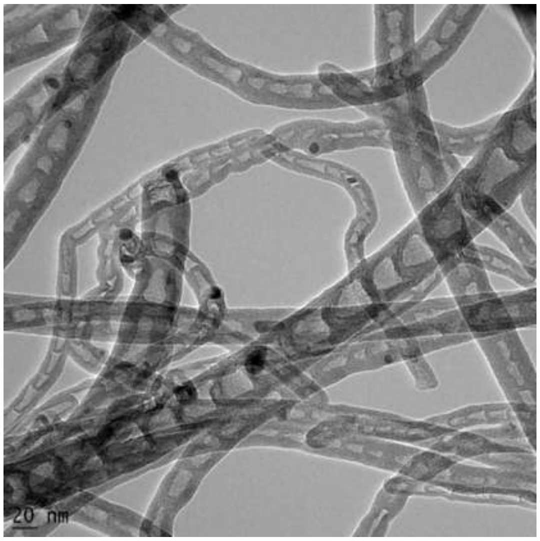

(a)

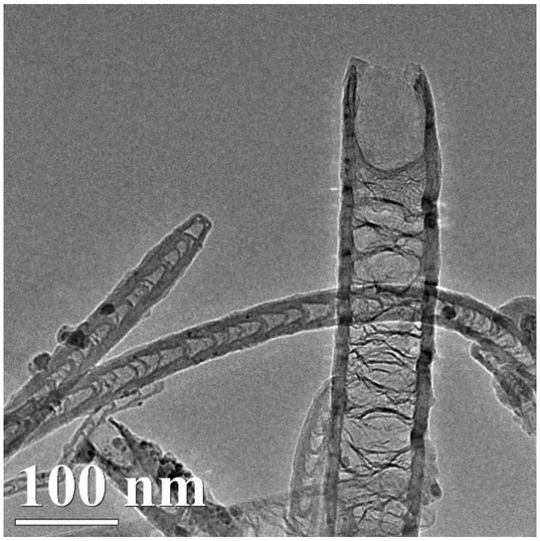

(b)

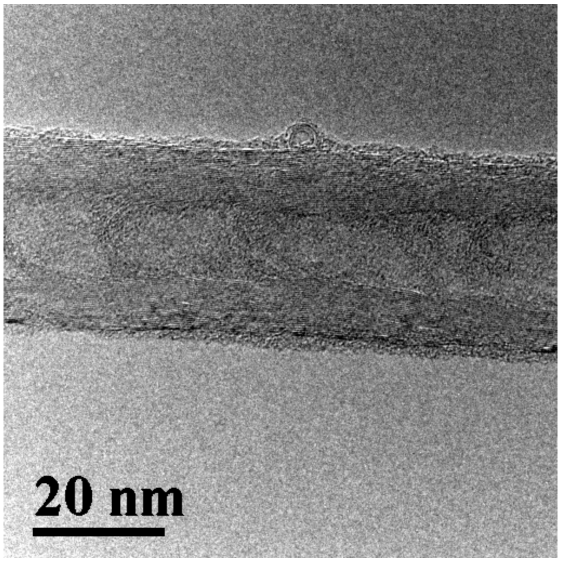

(c)

Figure S1. TEM images of as-prepared N-MWCNTs (**a**) and after treatment in HCl (**b**) as well as after irradiation by ion beam with φ_2=5.5 × 10^{16} ion·cm^{-2} (**c**).

Raman spectroscopy

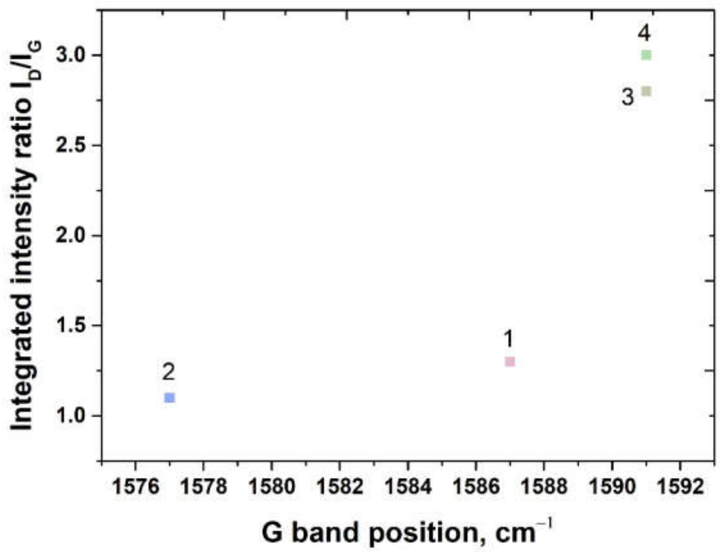

Figure S2. The G band position vs. I_D/I_G ratio for N-MWCNTs before and after treatments: 1 – initial; 2 – after HCl; 3 – after irradiation by ion beam with $\varphi_1=1.2 \times 10^{16}$ ion·cm^{-2}; 4 – after irradiation by ion beam with $\varphi_2=5.5 \times 10^{16}$ ion·cm$^{-2.}$

X-ray photoelectron spectroscopy

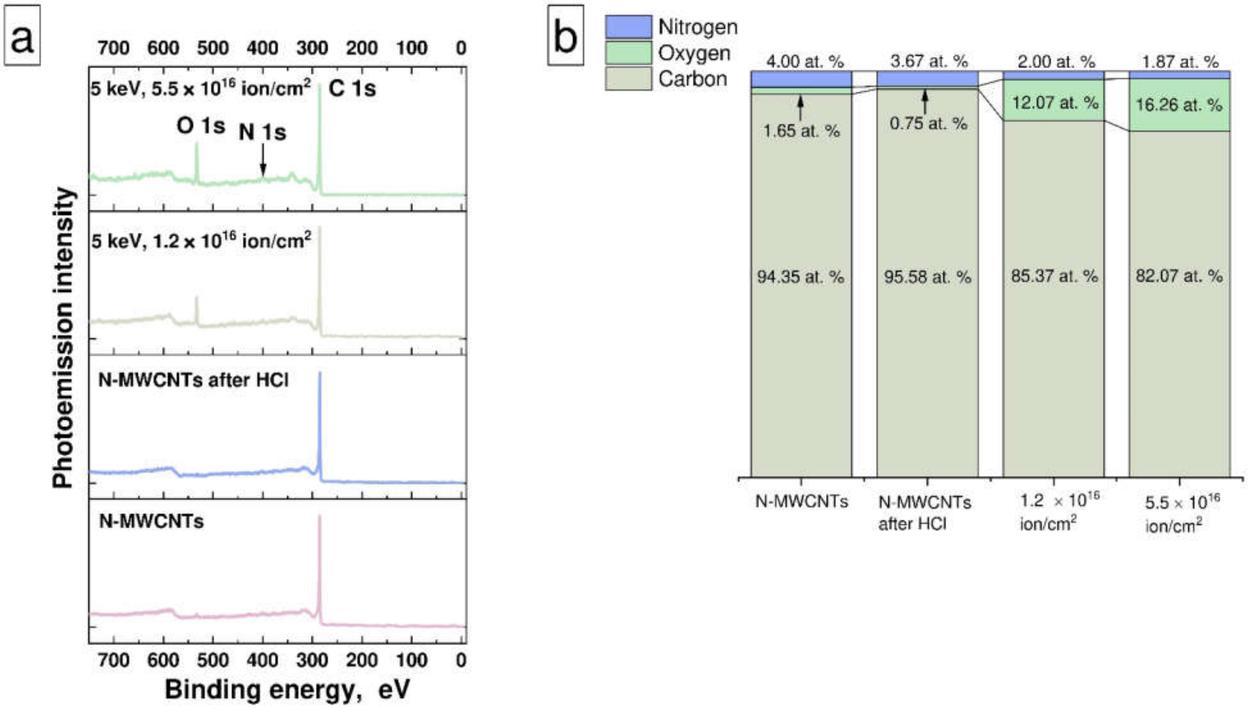

Figure S3. (**a**) Survey PE spectra of N-MWCNTs before and after treatments (hν=850 eV);.(**b**) diagram of atomic concentration from XPS survey spectra for samples.

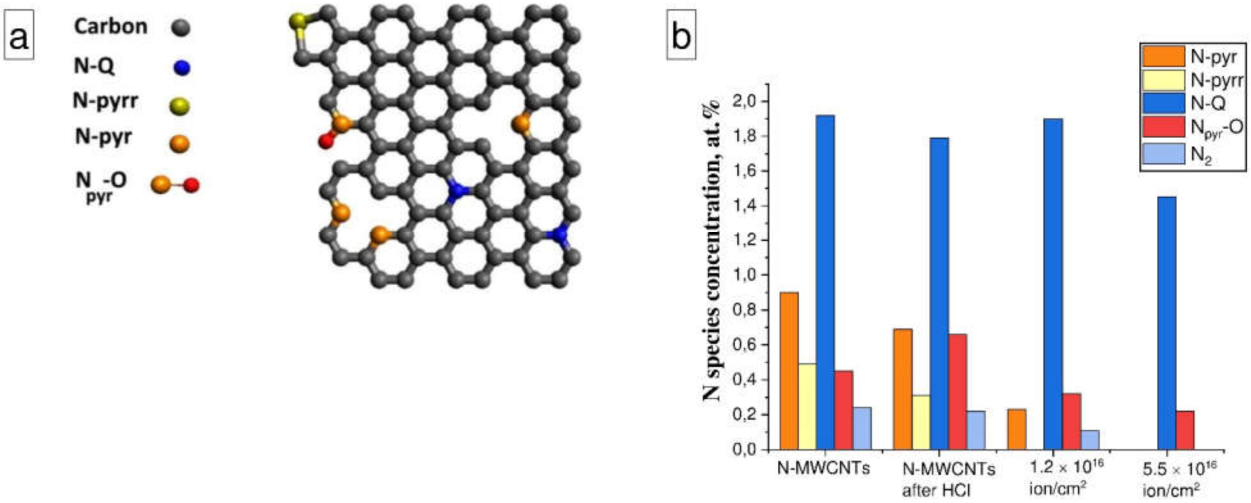

Figure S4. (**a**) Types of nitrogen inclusions in the hexagonal lattice of graphene; (**b**) the distribution of the concentrations of various nitrogen inclusions in N-MWCNTs before and after various treatments.

Electrochemical data obtained in 1M H_2SO_4 electrolyte

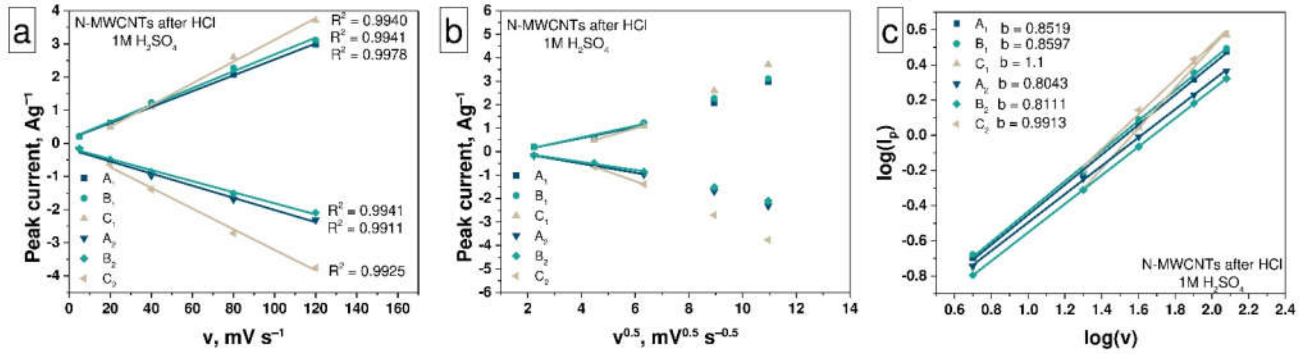

Figure S5. (**a**) Scan rate and (**b**) square root of scan rate vs peak current and (**c**) log of scan rate vs log of peak current plots for N-MWCNTs after HCl.

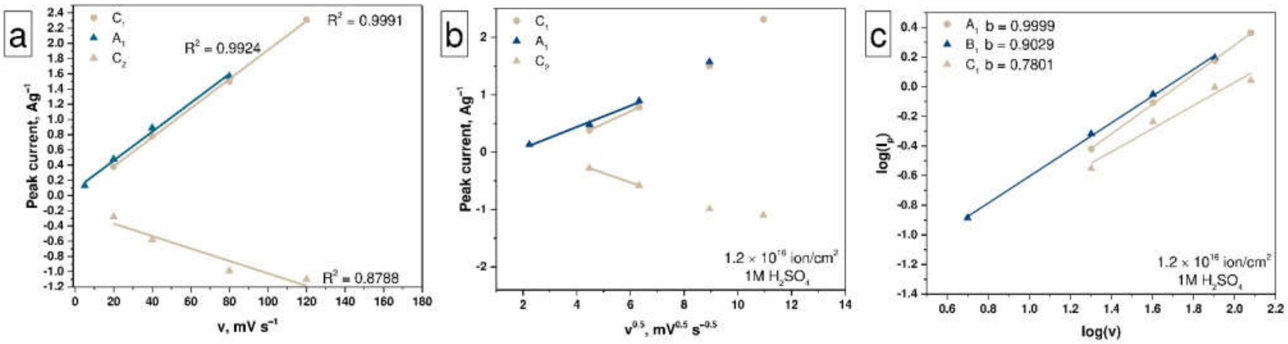

Figure S6. (**a**) Scan rate and (**b**) square root of scan rate vs peak current and (**c**) log of scan rate vs log of peak current plots for N-MWCNTs irradiated by ion beam with φ_1=1.2 × 10^{16} ion·$cm^{-2.}$

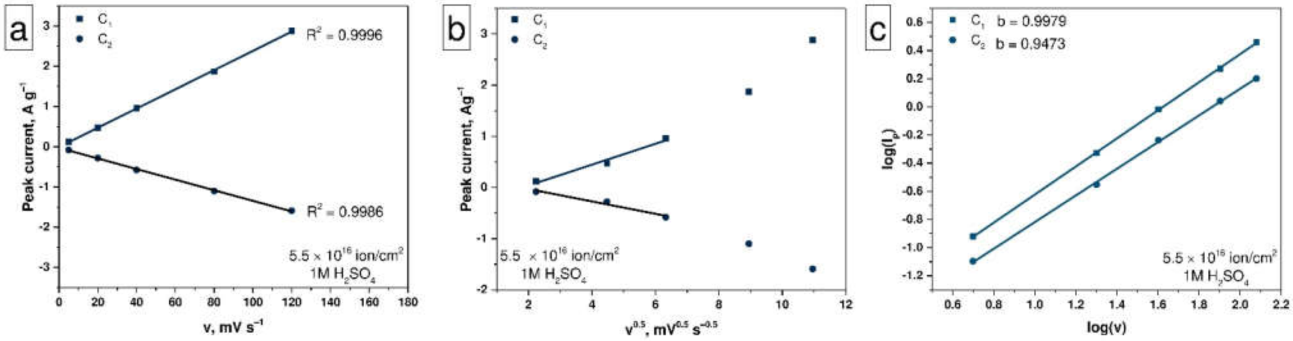

Figure S7. (**a**) Scan rate and (**b**) square root of scan rate vs peak current and (**c**) log of scan rate vs. log of peak current plots for N-MWCNTs irradiated by ion beam with φ_2=5.5 × 10^{16} ion·$cm^{-2.}$

Electrochemical and Raman data

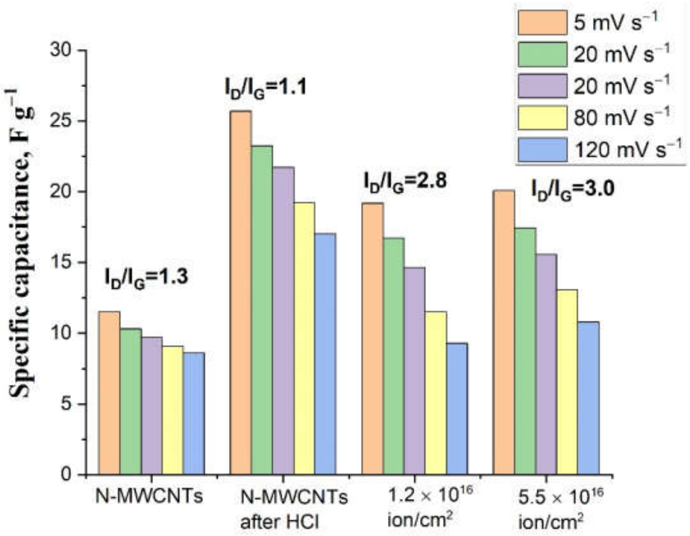

Figure S8. The diagram of the specific capacitance of nanotubes vs. their degree of defectiveness from the data of electrochemical measurements and Raman studies.
